# Five questions to consider when conducting COVID-19 phone research

**DOI:** 10.1136/bmjgh-2020-004917

**Published:** 2021-07-28

**Authors:** Shreya Menon, Petra Sonderegger, Swetha Totapally

**Affiliations:** Dalberg Global Development Advisors, Mumbai, Maharashtra, India

**Keywords:** COVID-19, public health, study design, qualitative study

**Summary box**

- Shifting one’s research modality from traditional face-to-face methods to phone surveys during the COVID-19 pandemic requires careful considerations of trade-offs.
- Our experience shows that before undertaking phone surveys, researchers need to clear a high bar of additionality, ensure their target populations can be representatively reached by phone, deploy short and clear surveys while putting in measures to reduce bias and increase response rates, minimise unique risks to respondents including around consent and distress response, and lastly be willing to invest what it takes to make stakeholders listen.
- An inability to meet these critical requirements should give researchers pause before conducting phone-based research.
- These findings and the evolving research on the effectiveness of different research modalities during the pandemic will likely hold implications for future research conducted by phone, especially in crisis contexts.

## Introduction

**Figure 1:**
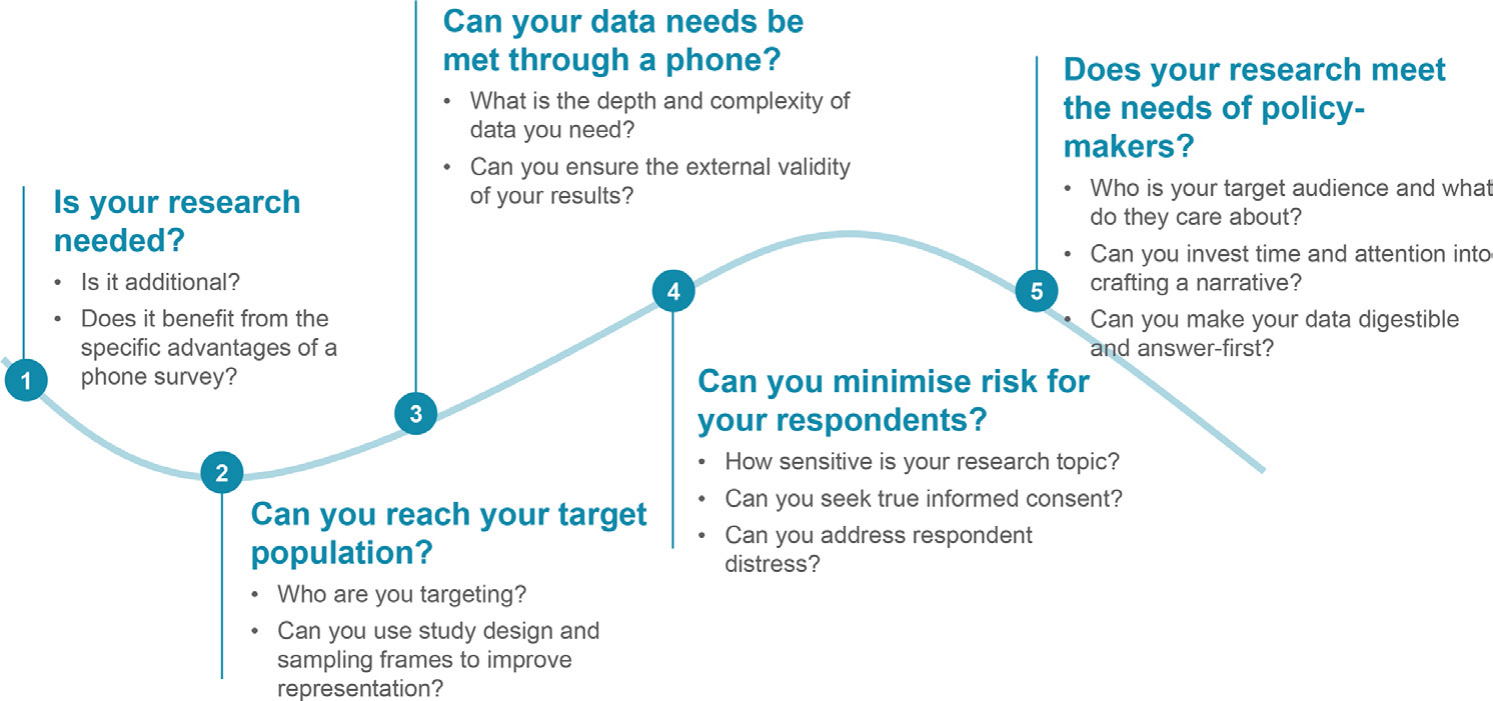
Five-part framework detailing the key questions and subquestions involved in deciding whether to undertake a phone-based survey.

The shift in research modality to phone surveys during the COVID-19 pandemic has highlighted tensions between the need for real-time data on the one hand and high-quality, generalisable data on the other. We present five questions for evaluating the trade-offs involved when navigating this shift (figure 1). We draw on prior research, our participation in the COVID-19 Research Network (CORE Net), and the range of phone research conducted by CORE Net from April to November 2020. The latter included four surveys across India on a range of topics and targeting diverse beneficiary populations. The largest of these was our survey from April-June 2020 to assess the efficacy of COVID-19-related governmententitlements across 47 000 low-income households in India. We also conducted asurvey of 17 000 women and men on the gendered impacts of Covid-19 fromOctober-November 2020, results of which are forthcoming. Additional CORE Netstudies referenced in this commentary include an ongoing study from April 2020by IDInsight on COVID-19 knowledge, behaviour change, and economic effects, aswell as a study from May-June 2020 by the International Food Policy andResearch Institute on incomes, livelihoods, and intra-household dynamics. In this commentary, we synthesise methodological learnings from the literature and researcher experiences during CORE Net’s surveys, and we draw examples from quantitative and qualitative work to provide a foundation-level understanding of when to do phone interviews and how to do them better. We consider the implications for phone surveys implemented during the COVID-19 pandemic and beyond, whether for public health research or other topics.

## 1. Is the research needed?

### Is it additional?

As with all research, additionality is important for phone surveys; calling people during a pandemic raises that bar significantly. In India alone, an estimated 1300+ primary research studies^1^ have been published since the pandemic began. Additionality becomes more important given this scale of data generation. Some tools that helped us clarify additionality include COVID-19-related survey archives (WHO, Azim Premji, Emerge), as well as collaboratives like CORE Net that allow organisations to coordinate research questions and share findings. For example, for their forthcoming studies on gender and health, respectively, Dalberg and IDInsight included identical questions on food security to enable comparisons across time and subpopulations.

Consider non-traditional additionalities. Studies that replicate a research question can still add value (eg, amplifying sample size, new demographic cuts). Qualitative interviews, although not generalisable, can nuance existing findings.

### Does it benefit from the advantages of phone surveys, for example, speed and scale?

Phone surveys are well suited to dipstick assessments of crises. Surveys like ours and Abdul Latif Jameel Poverty Action Lab’s (JPAL), which captured changes in access to government relief every 7–10 days, have helped direct relief efforts to specific districts and states.^2^ Similarly, a multiround survey by the National Council of Applied Economic Research that tested awareness of COVID-19 symptoms and support for lockdown was an excellent example of how telephonic interviews can be used as public health strategies evolve. Phone surveys have also enabled resource prioritisation in conflict zones and after natural disasters.^3^ Of course, speed and scale are also beneficial in non-crisis situations. For example, to complement their information management systems, Malawi’s health ministry used phone surveys to generate rapid data on their Integrated Community Case Management scheme and produce up-to-the-moment ‘snapshots’ to improve health service provision.^4^ Phone surveys can also be used to feed into rapidly evolving policy debates. For example, faced with rising public discontent, the Delhi Police is seeking to launch a feedback survey including 30 000 phone interviews.^5^

Studies that seek to assess a range of issues deeply and extensively should remain face-to-face. For example, the Demographic and Health Surveys Program suspended most fieldwork due to COVID-19-related travel restrictions.^6^

## 2. Can you reach the target population by phone?

### For population-wide studies

Random digit dialling (RDD) is adequate in most settings, although results may be biased by phone ownership rates. One study in Burkina Faso found a significant disparity in modern contraceptive use estimates between RDD and face-to-face surveys, even after applying poststratification weights.^7^ Under-representation can be mitigated through study design, for example, overcoming phone ownership disparity by asking to speak with randomly selected household members.^8^ Also consider adapting your messaging to your representativeness. For example, since our study under-represented agricultural households without phones, we framed findings as a ‘best-case scenario’.

### For research on populations with low access to phones (eg, women, rural households)

Private syndicated databases can help to better target a sample. However, their listings may be unrepresentative or have inbuilt biases. Matching enumerator and respondent characteristics can help. For example, IDInsight found that 13.8 percentage points more women completed their survey when the enumerator was female.^9^ Meeting respondents where they are is important. For example, our enumerators scheduled interviews with female respondents during hours when they had uninterrupted phone access after completing household chores.

### For research on niche populations (migrants, transgender individuals and so on)

Partner with organisations that work directly with your target group. The larger and more diverse your partner organisation’s base, the better. For example, Jan Sahas has a 60 000-strong base of migrants. Alternatively, quota-based convenience samples for hard-to-reach populations have achieved comparability with census characteristics.^10^ One COVID-19 study reported surmounting coverage and participation biases by combining randomly chosen numbers from a telecom database with recruitment via paid advertisements on social media.^11^

However, if you think your sampling limitations could discredit your findings, or if you need a pure random sample, consider pulling the plug.

## 3. Can your data needs be met through a phone call?

### What kind of questions are you asking?

Phone surveys are best suited for clear, simple questions with a low mental load. Complicated questions do not work, for example, Likert scales, 10-point scales, those that unfold from general to specific categories, or those that do not have clear endpoints and midpoints.^12^ Consider using simple semantic scales and shorter answer categories. Phone surveys are also more suited to impersonal questions; others may be listening in on your respondents’ answers and creating desirability bias.^13^ Experience from a phone-based assessment in Botswana shows that rapport is especially important in crisis times to ease respondent nervousness.^14^

That said, we successfully used telephonic human-centred design (HCD) interviews to supplement our quantitative findings. Our highly trained interviewers were able to conduct hour-long interviews and capture nuanced data by establishing a ‘platonic intimacy’ with respondents and reacting to subtle conversation cues to capture preferences, attitudes, coping strategies and more.

### Can you ensure external validity?

The physical absence of an enumerator in phone surveys can reduce certain biases; for example, respondents cannot infer an interviewer’s gender and other characteristics as easily. However, there is less control over the interview environment. The International Food Policy Research Institute’s (IFPRI) survey of front-line health workers found that 65% of female respondents were on speakerphone for at least some portion of their survey.^15^

High response and completion rates are integral to data quality. Tracking calls to build an optimal call-back protocol can help reach more respondents. Longer surveys can lead to lower response rates, so surveys should ideally be capped at 20 min. To cover additional content, we rotated survey sections between groups of respondents, but be cognisant that this may change the context of your questions and lead to a response effect.^16^ As mentioned above, matching enumerator and respondent characteristics also helps.

Errors can also result from incorrect use of computer-assisted telephone interviewing. Questions that require further probing or employ arithmetic checks increase the risk of error.^17^ As with all surveys, regular monitoring, feedback and standardisation are key. We found tools like JPAL’s checklist for transitioning to work-from-home phone surveying helpful.

Certain study designs cannot work in a telephonic context, for example, interviewing spouses simultaneously to understand intrahousehold dynamics, or using visual aids. If your research question needs these designs, or if you cannot invest in the training and measures required to achieve a minimum level of validity, perhaps a phone survey is not for you.

## 4. Can you minimise the risk to your respondents?

### What are you asking about?

When asking about issues like domestic violence, beware of unintended consequences, which are particularly difficult to mitigate over the phone. Female respondents may be at risk once the interview ends, or give low-fidelity answers. Sector experts with long-standing community ties may be best placed for such sensitive research. Helpful tools include the Data Collection on Violence against Women and COVID-19: Decision Tree.

### Can you gain true informed consent?

IFPRI’s experience with health workers showed that consent may be truer on a call since respondents feel less obliged to the interviewer, which may hold implications for improving the power dynamic between the interviewer and the respondent more generally.^18^ However, seeking consent remains difficult on the phone. We iterated extensively to simplify and standardise our oral consent script. In HCD interviews, we spent up to 20 min explaining consent to respondents and reiterating exit options where we gauged discomfort (one-word answers, long pauses). Using active, positive language (as opposed to passive voice) leads to higher-level consent.^19^

### Can you address respondent distress?

Phone surveys make it difficult to gauge distress. After first-hand experience in de-escalating a suicidal respondent, we retrained our enumerators to be more responsive and provide respondents with helpline information at the start of the interview before a potential dropoff, which we observed to have a reassuring effect.

If you cannot discharge your duty of care and manage the unique ethical risks of a telephonic survey, do not do one.

## 5. Does your research meet the needs of policymakers?

Actionability is often considered in terms of validity alone, but research has to meet a variety of requirements so that stakeholders can use it in their decision-making (especially in a crisis). We learnt the following from our own and others’ experiences. These lessons apply to many kinds of primary research, but are particularly relevant for phone research during a crisis, when time pressures and opportunity costs are high.

### Conduct research with a target audience in mind.

Who are you seeking to inform? Do they care about validity, demonstration effects, the political popularity of their decision or something else? Assessing what your stakeholders care about will help design a useful study. For example, one government stakeholder specified the exact p values and confidence intervals that would enable them to use our data, a triangulation point we used when defining our survey sample.

### Data alone are often not as persuasive to a policymaker as a clear, compelling narrative.

It is important to invest attention in crafting narratives that policymakers can use to both make decisions and justify them to others. Frank, in-depth discussions with stakeholders on the trade-offs, risks and effects of using data tend to increase the use of that data.^20^

### Try to present digestible results, answer-first.

Many of our government stakeholders were in a war-room mode and did not have time to read detailed reports. Early findings communicated informally over WhatsApp proved more useful. Another government stakeholder told us that while a telephonic survey was not good enough for their audit, it would be useful directionally. We therefore oriented our presentation towards recommendations instead of focusing on methodology.

If you cannot invest the resources in seeing the impact of your research from end to end, consider finding a partner to support you or putting off your research until you are able to do so.

## Conclusion

For years, many have observed the potential for phone surveys to enable speedy research. As the COVID-19 crisis unfolds, we will gain richer information on the limitations of phone surveys and how to work around them—learnings that can help improve our research in normal times as well. Our hope is that when another crisis hits in the near or distant future, the health research community will find itself better placed to collect and disseminate actionable evidence.

## Data Availability

Data are available upon request
